# Smokescreen: a targeted genotyping array for addiction research

**DOI:** 10.1186/s12864-016-2495-7

**Published:** 2016-02-27

**Authors:** James W. Baurley, Christopher K. Edlund, Carissa I. Pardamean, David V. Conti, Andrew W. Bergen

**Affiliations:** BioRealm LLC, 6101 W. Centinela Ave., Suite 270, Culver City, CA 90230-6359 USA

**Keywords:** Addiction, Nicotine dependence, Nicotine metabolism, Pharmacogenomics, Smoking cessation, Genome-wide association study, Bioinformatics, Biomarkers

## Abstract

**Background:**

Addictive disorders are a class of chronic, relapsing mental disorders that are responsible for increased risk of mental and medical disorders and represent the largest, potentially modifiable cause of death. Tobacco dependence is associated with increased risk of disease and premature death. While tobacco control efforts and therapeutic interventions have made good progress in reducing smoking prevalence, challenges remain in optimizing their effectiveness based on patient characteristics, including genetic variation. In order to maximize collaborative efforts to advance addiction research, we have developed a genotyping array called Smokescreen. This custom array builds upon previous work in the analyses of human genetic variation, the genetics of addiction, drug metabolism, and response to therapy, with an emphasis on smoking and nicotine addiction.

**Results:**

The Smokescreen genotyping array includes 646,247 markers in 23 categories. The array design covers genome-wide common variation (65.67, 82.37, and 90.72 % in African (YRI), East Asian (ASN), and European (EUR) respectively); most of the variation with a minor allele frequency ≥ 0.01 in 1014 addiction genes (85.16, 89.51, and 90.49 % for YRI, ASN, and EUR respectively); and nearly all variation from the 1000 Genomes Project Phase 1, NHLBI GO Exome Sequencing Project and HapMap databases in the regions related to smoking behavior and nicotine metabolism: *CHRNA5-CHRNA3-CHRNB4* and *CYP2A6-CYP2B6.* Of the 636 pilot DNA samples derived from blood or cell line biospecimens that were genotyped on the array, 622 (97.80 %) passed quality control. In passing samples, 90.08 % of markers passed quality control. The genotype reproducibility in 25 replicate pairs was 99.94 %. For 137 samples that overlapped with HapMap2 release 24, the genotype concordance was 99.76 %. In a genome-wide association analysis of the nicotine metabolite ratio in 315 individuals participating in nicotine metabolism laboratory studies, we identified genome-wide significant variants in the *CYP2A6* region (min *p* = 9.10E-15).

**Conclusions:**

We developed a comprehensive genotyping array for addiction research and demonstrated its analytic validity and utility through pilot genotyping of HapMap and study samples. This array allows researchers to perform genome-wide, candidate gene, and pathway-based association analyses of addiction, tobacco-use, treatment response, comorbidities, and associated diseases in a standardized, high-throughput platform.

**Electronic supplementary material:**

The online version of this article (doi:10.1186/s12864-016-2495-7) contains supplementary material, which is available to authorized users.

## Background

Addictive disorders represent debilitating conditions that result in productivity loss, and an increased risk for associated mental disorders as well as infectious, metabolic, proliferative, respiratory, and vascular diseases [1]. Addictions encompass substance-use disorders and compulsive behaviors [2]. The heritabilities of substance-use disorders are consistently found to be ~50 % [3] with the lowest heritability for hallucinogen and highest for cocaine use disorders, respectively [4]. The ~2:1 monozygotic:dizygotic twin concordance ratios for many substance-use disorders support additive genetic effects and multiple loci [4]. Estimates for the influence of genome-wide common variants on nicotine and alcohol dependence, and illicit drug use traits are ~30–36 % [5, 6], representing most of the estimated heritability. There is evidence for shared genetic influence across multiple substance-use disorders [6, 7].

Among all substance-use disorders, smoking is the leading cause of preventable death in the United States and is associated with cancer, aerodigestive tract, genitourinary tract, and vascular diseases [8]. Nicotine dependence is most often assessed using measures of cigarette consumption (cigarettes per day, CPD) or dependence (Fagerström Test for Cigarette Dependence, FTCD) [9, 10]. Genetic influences predominate over environmental influences in smoking initiation, but shared environment is important during the adolescent period when the majority of future smokers initiate smoking [11]. Developmental analyses suggest that genetic and environmental factors that influence risk for smoking initiation and consumption are independent in adolescence, but become correlated in emerging adults [12]. Genome-wide meta-analyses of cigarette consumption, dependence, and exposure measures, including linkage [13, 14] and association [15–22] studies, have focused attention on multiple regions, including specific variants in cholinergic, cytochrome oxidase, dopaminergic, and hypoxia response genes.

People who quit smoking reduce their risk of disease and those who quit at an earlier age see the most health benefits [23]. An estimated 69 % of the approximately 45 million smokers in the United States want to stop smoking [24, 25]. Nonetheless, smoking cessation success rates remain discouragingly low; in 2010, only 6.2 % of adult smokers attempting to quit were successful [25]. For those seeking to quit, there are a variety of counseling methods and cessation medications available [26, 27].

Genetics plays a role in smokers’ cessation attempts (heritabilities of ~50 % [28, 29]), and response to cessation treatments [30]. Pharmacogenetic analyses of smoking cessation clinical trials suggest that prospective abstinence is affected by loci influencing reward system responses to nicotine and pharmacotherapeutics, nicotine and bupropion metabolism, and varenicline clearance [30]. Analyses of chr15q25.1 SNPs and prospective abstinence by pharmacotherapy have been mixed: null [31–33], reduced in participants randomized to placebo [34], and increased in participants randomized to multiple therapies [35, 36]. In the largest analysis to date [37], chr15q25.1 smoking-heaviness risk SNPs were found to be associated with reduced abstinence in participants randomized to placebo, and increased abstinence in participants randomized to nicotine replacement therapy (NRT). In contrast, studies on functional variation in the nicotine metabolizing enzyme cytochrome P450 oxidase 2A6 (*CYP2A6*) [38, 39] translate robustly to smoking behaviors [40, 41] and prospective abstinence, using either nicotine metabolite [42–45], or genetic [46–48] analyses. In brief, recent findings suggest that slow nicotine metabolizers are less nicotine dependent and have similar quit rates across therapies, while fast nicotine metabolizers are more nicotine dependent and may benefit from combined treatments with NRT, bupropion, or varenicline.

Despite these successes, attempts to individualize therapies using genetics have been limited by inconsistent results. Existing evidence suggests that smoking cessation prediction is influenced by not just genetics, but also by patient characteristics (gender, age of onset, nicotine dependence, and race/ethnicity) and treatment protocol (clinician interaction; type, number and length of counseling sessions; and pharmacotherapy). Biomarkers, specifically the nicotine metabolite ratio (NMR, the ratio of 3’-*trans*-hydroxycotinine/cotinine), have also been shown to influence pharmacological therapies success [42, 43, 45]. Advancing the understanding of complex relationships among multiple genetic and environmental factors, smoking behavior, nicotine dependence, and treatment outcomes requires large sample sizes. Current clinical studies are challenged by relatively small sample sizes in treatment arms; thus, pooling data across clinical trials and observational studies is essential, but complicated by heterogeneity in trial study designs, genotyping technologies, and genetic marker content.

Motivated by these obstacles, we developed the Smokescreen genotyping array, a research tool for significantly advancing the understanding of addiction and the development of predictive models that can potentially be used to personalize treatment strategies for addiction, including nicotine- and tobacco-related outcomes.

## Results

### Array design and coverage estimates

The final array design included 646,247 markers: 296,038 genome-wide association markers; 255,862 tag SNPs and 17,632 exonic markers in addiction-related gene regions; 11,099 fine-mapping markers in loci related to nicotine metabolism and smoking behavior; and the remaining markers in other categories (Table 1; markers in categories have some overlap). Coverage estimates are provided using the number of variants available through genotype imputation in the 1000 Genomes Project Phase 1 [49] Yoruba in Ibadan, Nigeria (YRI), East Asian (ASN), and European (EUR) populations (Table 2).

**Table 1:** Smokescreen genotyping array content

Category	Markers^a^
*1014 addiction-related genes*	
Tag SNPs (MAF ≥ 0.05)	255862
Exonic markers	17632
*Genome-wide association markers*	
Affymetrix’ Axiom® Biobank GWAS grid	246038
African (YRI) booster panel	50000
*Fine-mapping of smoking related loci*	
*CHRNA5*-*CHRNA3*-*CHRNB4* (552 kb LD block)	8913
*CYP2A6* (±20 kb)	573
*CYP2B6* (±20 kb)	1613
*High-value addiction markers*	
NeuroSNP Project	4994
Pharmacogenetics of Nicotine Addiction Treatment (PNAT) SNP panels	2271
v1.0 Quit Success Score	12058
Literature search	1329
*Comorbidity markers*	
Lung Cancer	3091
Psychiatric disorders	1200
Tobacco smoke constituent update and metabolic phenotypes	1907
Pulmonary diseases and traits	7945
Cardiovascular diseases and traits	2247
*General high-value markers*	
Pharmacogenomic markers	2030
NHGRI GWAS Catalog	7612
eQTLs	9736
Loss-of-function markers	4680
Ancestry informative markers (AIMs)	5545
HLA/KIR	8894
Mitochondrial	180
Array Total	646247

^a^Markers in categories may overlap

**Table 2:** Counts of imputed SNPs (1000 Genomes Project: Phase 1, March 2012 release)

	YRI	ASN	EUR
Genome-Wide			
MAF ≥ 0.01	15263433	8091434	9213645
MAF ≥ 0.05	9219112	5973609	6505846
Addiction Genes			
MAF ≥ 0.01	794696	417902	476661
MAF ≥ 0.05	474408	303991	333356
*CHRNA5*, *CHRNA3* and *CHRNB4*			
MAF ≥ 0.01	3377	2377	1922
MAF ≥ 0.05	2121	1862	1519
*CYP2A6* and *CYP2B6*			
MAF ≥ 0.01	2886	2679	2696
MAF ≥ 0.05	2157	2043	2136

#### Genome-wide association markers

**Fig. 1:**
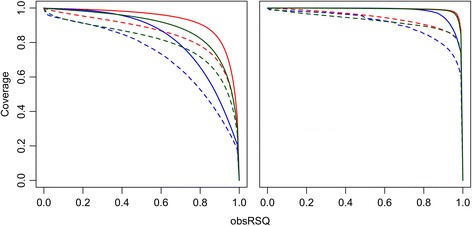
Smokescreen coverage estimates by increasing observed imputation *r* ^2^ thresholds: genome-wide and addiction genes. *Left panel*: Genome-wide coverage. *Right panel*: Addiction genes coverage. *Solid line*: MAF ≥ 0.05. *Dashed line*: MAF ≥ 0.01. *Red*: EUR. *Blue*: YRI. *Green*: ASN. Observed imputation *r* ^2^ (obsRSQ) is the correlation between imputed (continuous) genotype dosage and the measured genotype from the 1000 Genomes Project. The proportion of 1000 Genome Project Phase 1 variants with an obsRSQ above the threshold on the *x*-axis is represented on the *y*-axis. The average obsRSQ differs by race/ethnicity and by array content categories (e.g., genome-wide versus addiction genes). The coverage (fraction of variants with obsRSQ above the threshold) decreases as the obsRSQ threshold increases. A typical threshold used in evaluating array coverage is 0.80

The array includes 296,038 markers for genome-wide coverage of common genetic variations. The relationship between genome-wide array coverage and the observed correlation of imputed and actual genotypes (obsRSQ) in 1000 Genomes Phase 1 YRI, ASN, and EUR populations is shown in Fig. 1 (*left panel*). As the threshold on obsRSQ increases (*x*-axis), fewer variants exceed the threshold, and the coverage decreases (*y*-axis). The array design achieves good coverage of common variants (MAF ≥ 0.05, obsRSQ > 0.8): 65.67, 82.37, and 90.72 % in YRI, ASN, and EUR populations respectively, and covers 52.71, 71.68, and 78.17 % respectively of variants with MAF ≥ 0.01. The average obsRSQ for variants with MAF ≥ 0.05 were 0.82, 0.89 and 0.93 for YRI, ASN, and EUR respectively, and 0.72, 0.80, and 0.85 for variants with MAF ≥ 0.01. These metrics demonstrate the array’s suitability for genome-wide analyses and meta analyses based on genotype imputation using 1000 Genomes Project Phase 1 data.

#### Addiction genes

The array contains 273,494 markers for dense coverage of 1014 genes (±20 kb) across populations. The array captures 97.47, 98.09, 98.08 % of common variants (MAF ≥ 0.05) and 63.61 %, 84.02, and 82.55 % of MAF ≥ 0.01 variants in YRI, ASN, and EUR populations, respectively, directly through linkage disequilibrium (pair-wise *r*^2^ ≥ 0.8). The fraction of variants covered by genotype imputation in these gene regions for YRI, ASN, and EUR populations is shown in Fig. 1 (*right panel*). Consequently, the array has outstanding imputation coverage (98.72, 99.31, and 99.43 %) of common variants and excellent coverage of MAF ≥ 0.01 variants: 85.16, 89.51, and 90.49 % for YRI, ASN, and EUR respectively (obsRSQ ≥ 0.8). In the same order, average obsRSQ of all variants within the addiction gene regions were 0.98, 0.99, and 0.99 for common variants, and 0.91, 0.92, and 0.94 for MAF ≥ 0.01 variants, indicating that imputation works well for most variants in these regions. The list of 1014 genes and each region’s imputation metrics are provided in Additional file 1.

#### Fine-mapping

Eight thousand, nine hundred and fourty-eight SNPs and indels (average of 1 marker per 62 base pairs) were selected for the 552 kb LD block encompasing the chr15q25.1 nicotinic acetylcholine receptor (nAChR) gene cluster (CHRNA5, CHRNA3 and CHRNB4). For *CYP2A6* (±20 kb), 612 markers were selected with an average of 1 marker every 75 base pairs. For *CYP2B6* (±20 kb), 1628 markers were selected with 1 marker per 45 base pairs on average. As expected, the imputed coverage and average obsRSQ was greater than 99 % in the 1000 Genomes Project Phase 1 YRI, ASN, and EUR populations, regardless of minor allele frequency. Additional markers were selected to capture variation in the surrounding region, including *EGLN2*, *CYP2A7*, *CYP2G1P*, and *CYP2B7P1*.

#### Additional content

Additional markers were included in the design for compatibility with consortium-developed arrays and most recent findings (Table 1). The array contains: 2271 markers from the candidate gene/pathway arrays developed and used by the Pharmacogenetics of Nicotine Addiction Treatment (PNAT) research program [31, 37]; 3091 markers from a lung cancer meta-GWAS [50]; 1200 markers related to psychiatric comorbidities from the Psychiatric Genetics Consortium [51]; and 7956 and 2247 markers for pulmonary and cardiovascular phenotypes respectively from the UK Biobank Axiom Array [52].

The array also includes: 1329 markers related to addiction identified in recent literature from NIDA Genetics Consortium investigators; 12,058 markers used in the smoking cessation v1.0 Quit Success Score biomarker [53]; 2030 pharmacogenomic markers related to absorption, distribution, metabolism, and excretion (ADME); and 7612 markers identified in previous genome-wide association studies or addiction and related diseases [54]. The array includes a panel of 5525 ancestry informative markers (AIMs) for ancestry estimation and evaluation of population substructure.

### Genotyping quality

From 636 samples derived from blood or cell line, 622 (97.80 %) passed quality control based on recommended best practices for Axiom arrays by Affymetrix [55]. Several samples were excluded from subsequent analyses; one sample failed to scan, eight had Dish QC < 0.82 due to a sample processing issue, and five had stage one genotype call rates < 97 %. In addition, seven negative controls were processed across the genotyping plates, and all exhibited low separation from the background signal as expected. In passing samples, 582,143 (90.08 %) of the markers on the array passed quality control, using Affymetrix recommended best practices. With default settings of Affymetrix SNPolisher classifications, we removed: 48,083 markers classified as “Other”; 7014 classified as “CallRateBelowThreshold”; 1765 classified as “Off-target variant (OTV)”; 181 classified as “Hemizygous”; and 7061 classified as “MonoHighResolution” that also had previously failed Affymetrix’s internal validation process (see Table 3).

**Table 3:** SNP classifications and recommendations using 622 passing samples derived from blood or cell line

SNPolisher ConversionType	Previously validated markers	Previously failed-validation markers	De novo markers	Total SNPs	Recommendation to keep for analysis
PolyHighResolution	441120 (68.26 %)	17595 (2.72 %)	7547 (1.17 %)	466262 (72.15 %)	yes
NoMinorHom	62639 (9.69 %)	6360 (0.98 %)	6173 (0.96 %)	75172 (11.63 %)	yes
MonoHighResolution	12095 (1.87 %)	7061 (1.09 %)	28614 (4.43 %)	47770 (7.39 %)	yes if previously validated or de novo
Other	6031 (0.93 %)	14515 (2.24 %)	27537 (4.26 %)	48083 (7.44 %)	no
CallRateBelowThreshold	3078 (0.48 %)	2856 (0.42 %)	1080 (0.17 %)	7014 (1.09 %)	no
OTV	370 (0.06 %)	535 (0.08 %)	860 (0.13 %)	1765 (0.27 %)	yes if off-target variant genotyped
Hemizygous	180 (0.03 %)	1 (<0.01 %)	0 (0.00 %)	181 (0.03 %)	yes if visually inspected
TOTAL	525513 (81.32 %)	48923 (7.57 %)	71811 (11.11 %)	646247	

‘*Previously validated*’ or ‘*Previously failed-validation*’ are markers tested by the manufacturer. ‘*De novo*’ are markers on the array but not validated by the manufacturer. *‘PolyHighResolution’* and *‘NoMinorHom’* are markers with good cluster resolution. *‘MonoHighResolution’* indicates that fewer than two examples of the minor allele was present. ‘*CallRateBelowThreshold’* indicated that the SNP call rate is below the threshold while other properties are above the threshold. *‘Other’* are markers where one or more cluster properties falls below its threshold

The average genotype reproducibility in 25 replicate pairs across all passing SNPs was 99.94 %. Of the passing markers, 40,745 (7.00 %) were monomorphic, 17,099 (2.94 %) had minor allele frequency (MAF) greater than 0 and less than 0.01; 125,359 (21.53 %) had MAF between 0.01 and 0.05; 398,940 (68.53 %) had MAF greater than or equal to 0.05. For 137 HapMap samples [56] (45 JPT, 32 CEU, 60 YRI) and 226,511 passing markers that overlapped with HapMap2 release 24 (marker call rate ≥95 % in HapMap2), the average genotype concordance was 99.76 %.

### Smokescreen application to nicotine metabolism analysis in multiple populations

Unrelated African American (*N* = 52), Asian American (*N* = 55), and European American ancestry individuals (*N* = 239) from three existing laboratory-based nicotine metabolism studies were selected for genotyping on the Smokescreen array [57]. For all samples, the NMR is defined as the *trans*-3’-hydroxycotinine to cotinine ratio. In these three studies, biospecimens were collected, and creatinine-adjusted cotinine and *trans*-3’-hydroxycotinine levels were estimated via mass spectrometry using an established method [57].

In the Pharmacokinetics in Twins (“PKTWIN”) [58] study, participants were recruited from the Northern California Twin Registry in a multiple stage protocol to coordinate ascertainment of twins to investigate heritability and genetic components of nicotine metabolism. Participants consented to a 30-min venous administration of nicotine and cotinine, followed by an 8-hour hospital stay for blood and urine biosample collection. In the Pharmacogenetic Study of Nicotine Metabolism (“588”) [57], recruiting by smoking status and gender of European, African, and Asian Americans was performed through multi-media advertisements for a nicotine and cotinine metabolism study. Participants consented to morning oral administration of nicotine, and either labeled (smokers) or unlabeled (nonsmokers) cotinine. The following biospecimens were collected: saliva up to 60 h after dosing; blood up to 480 min; urine up to 8 h. In the Smoking in Families (SMOFAM) study [59], individuals from 61 pedigrees with at least three ever-smokers individuals per pedigree originally recruited to assess the relations among genetic factors, environmental factors, and tobacco use, consented to oral administration of a fixed dose of nicotine and cotinine at home monitored by a nurse, followed by salivary sample collection at multiple time points as well as a blood sample for DNA analysis [57].

**Fig. 2:**
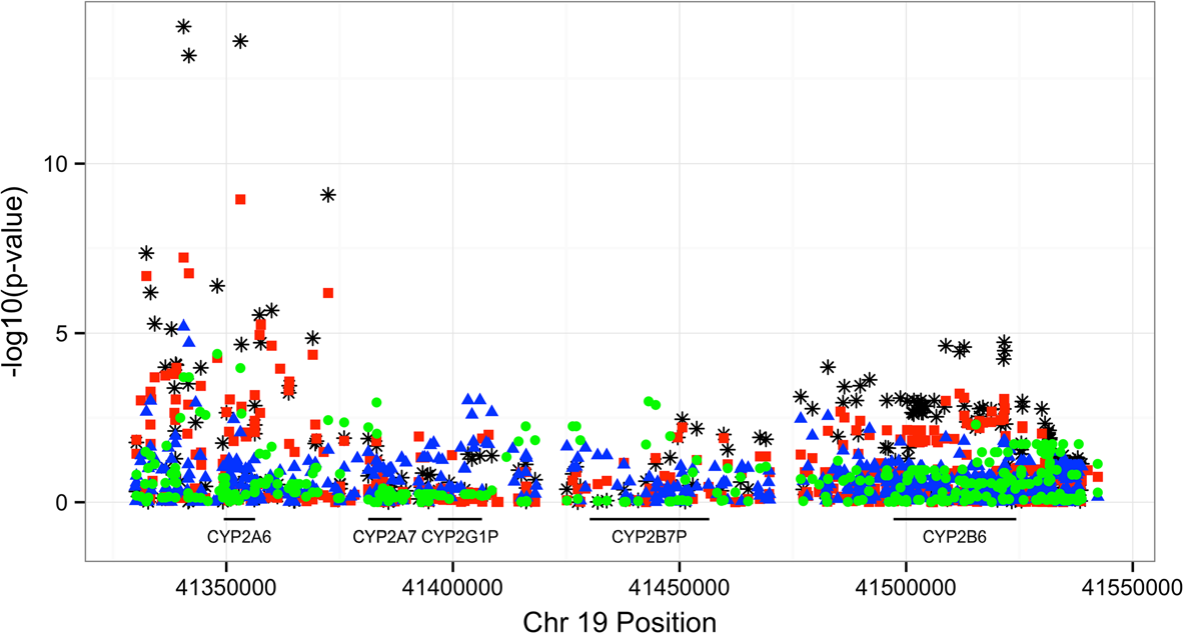
*CYP2A6* - *CYP2B6* regional association with the nicotine metabolism ratio. *Blue triangle*: African-American. *Green circle*: Asian-American. *Red square*: European-American. *Black star*: Meta-analysis

Three hundred fifteen samples passed quality control with complete data and were included in the analyses (from 49 African, 51 Asian, and 215 European American participants). Multiple factors including population structure principal components, age, sex, BMI, and smoking status were incorporated to model NMR in a genome-wide, multi-ethnic meta-analysis. Adequate Type I error control was observed with genome-wide significant results at *CYP2A6* (min *p* = 9.10E-15). The top SNP accounts for 12 %, 27 %, and 19 % of the NMR variation in European Americans, African Americans, and Asian Americans, respectively. The minimum *p*-value in the nearby *CYP2B6* region was 1.85E-5 with association patterns differing by ancestry (Fig. 2). Association patterns with the NMR remained intact for *CYP2B6* in an analysis adjusting for *CYP2A6*’s top SNP; other *CYP2A6* SNPs showed evidence of independent association after this adjustment (min *p* = 1.71E-7).

## Discussion

Until recently, manufacturing a genotyping array, especially with primarily custom-content, was prohibitively expensive. The Smokescreen array represents the next generation of targeted arrays, providing both baseline genome-wide content, and enhanced coverage of important regions and pathways specific to a phenotype or group of related phenotypes. The popularity of these similarly-sized arrays can be seen in the Psychiatric Genetics Consortium Infinium PsychArray [60] with 571,054 SNPs (271,406 genome-wide tag SNPs, 276,701 exonic markers, and ~50,000 markers associated with common psychiatric disorders) and the OncoArray Consortium OncoArray-500 K [61] with 499,170 SNPs (275,691 genome-wide tag SNPs and 223,479 cancer specific SNPs). These three arrays offer a core set of markers that provide similar imputation-based genome-wide coverage. Used a procedure similar to Nelson et al. [62], we estimated that the Smokescreen design covers 66 %, 82 %, and 91 % of common variants (MAF ≥ 0.05) in YRI, ASN, and EUR, respectively. The Smokescreen array includes extensive custom content (350,209 markers), focusing on enhancing coverage of gene regions and pathways related to addiction (dependence, drug metabolism, and treatment response), and attributable disease (proliferative, psychiatric and pulmonary outcomes) in multiple populations. Consortia developed arrays have similar goals: to provide content of value to researchers studying related phenotypes and to provide data for large meta-analyses and replication studies.

Genome-wide arrays, such as Smokescreen, allow for imputation of common and rare genotypes using haplotype reference panels. Recent haplotype projects (e.g., the Haplotype Reference Consortium, which combines multiple cohorts including the 1000 Genomes Project) allow for more accurate imputation of less common and rare variants [63, 64]. The ability to genotype large numbers of study samples and impute more variants accurately, enables powerful meta-GWAS studies [65]. Additionally, genome-wide imputation allows for discovery of associations in regions not directly enriched for in the array design. This is important as meta-analysis consortia and multiple methods [51] identify additional addiction-related regions.

There are some limitations in the estimation of Smokescreen array performance. It should be noted that the coverage is an estimate based on the design of the array. The realized coverage is not yet computable as the validation of markers is ongoing. Approximately 12 % of SNPs on the array are too rare to be observed with the current sample size. Additional samples will improve genotype clustering of rare SNPs or SNPs with low quality metrics. Some pilot samples derived from saliva were genotyped, but the sample size was not sufficient for estimating performances on these samples separately. In general, we recommend saliva samples to be collected using kits with preservatives. Laboratories familiar with Axiom Arrays should assess the quality of salivary samples with suspect collection or storage method prior to genotyping.

Ascertainment biases in the markers selected on the array or the reference population used in imputation may lead to lower coverage, as commonly observed in African ancestry populations. However, increasing coverage on the array (as we did with the African (YRI) booster panel) and diverse reference panels for imputation, helps mitigate this issue. Another limitation of these approaches over sequencing, is the identification of *de novo* mutations. These mutations, however, account for a small fraction of both rare and common neurodevelopmental diseases and require pedigrees for analysis [66].

We envision the Smokescreen array driving translational research by facilitating the development of algorithms, derived from multiple genetic and clinical factors for risk prediction and treatment approach assignments. Previously, genome-wide allelotyping analyses of smoking cessation trials revealed associations of common variants with prospective abstinence [67]. This research lead to the design of a clinical trial analysis model incorporating a “quit-success” genetic score, which retrospectively predicted abstinence in a randomized trial stratifying smokers by nicotine replacement therapy dose and dependence [53]. This model used both genetic (“quit-success” score) and clinical (FTCD score) information. We re-envision this model based on a Smokescreen analysis platform that incorporates individual level genotype data, additional clinical factors, and the multi-stage process of validation and utility assessment in large sample sizes derived from meta-analysis of multiple trials [68]. For example, genotyping samples with multiple addiction-related phenotypes will permit genome-wide correlation [69–71] and estimation of the extent of shared variance and polygenicity among dependence, attributable disease, and treatment response; the proportion of shared variance among dependencies using genome-wide correlation is substantial [6].

The addiction-related gene content of Smokescreen is designed to be helpful in pharmacogenetic analyses of current or future addiction gene targets. In an analysis of the Psychiatric Genetic Consortium schizophrenia findings [72], 40 of the 341 protein-coding genes linked to GWAS hits were identified as targets of existing drugs or drugs undergoing Phase III trials [73]. Lencz and Malhotra conclude that six protein-coding genes (*CACNA1C*, *CACNB2*, *CACNA1I*, *DRD2*, *GRIN2A*, *HCN1*) are of greatest neuropsychiatric and genetic interest. Three of these six genes (*DRD2*, *CACNA1I*, *GRIN2A*) are included in Smokescreen’s group of addiction-related genes; overall, 10 of the 40 target genes are included in Smokescreen’s gene list with increased coverage, suggesting Smokescreen’s usefulness for addiction-related drug development studies. Coverage of the nicotine metabolizing enzyme genes *CYP2A6* and *CYP2B6*, the opportunity to incorporate NMR measures in larger studies, and novel analytical methods may improve nicotine metabolism models, which currently predict between 50 and 70 % of variance in European ancestry populations [39].

## Conclusions

The Smokescreen array achieves robust genome-wide coverage of common variants, and exceptional coverage of 1014 genes relevant to addiction and known nicotine metabolism and smoking behavior regions in African (YRI), Asian, and European populations. These, paired with content from recent findings and related work in pharmacogenomics, comorbidities, and attributable diseases create a comprehensive genotyping array for addiction research. Analytical validity and utility were demonstrated through pilot genotyping of HapMap and study samples. For HapMap samples, the genotype concordance for overlapping content was > 99 %. Based on samples from nicotine metabolism laboratory studies, we identified variants at genome-wide significance in a region known to be highly influential on nicotine metabolism, serving as a positive confirmation of the array’s design. These attributes enable researchers to perform genome-wide, candidate gene, and pathway-based association analyses on various addictions, including those related to smoking and tobacco use.

## Methods

### Array design objectives

The array design was driven by the need for a common panel of markers for both hypothesis-driven and genome-wide studies of addiction. This project was funded by the Small Business Innovative Research (SBIR) program with the National Institute on Drug Abuse (NIDA). Specific requirements included capturing prioritized markers identified by NIDA; selecting focused content for a wide variety of research purposes and multiple ethnicities; selecting a platform for accurate and reproducible data across studies, and providing a screening tool for development of risk assessments and personalized approaches to addiction treatment.

The ability to customize the array was important to cover common and rare variation across populations genome-wide, with enhanced coverage in genes and regions related to addiction. Manufacturing reproducibility was also critical for translational and clinical use-cases that may be developed based on the array. Given these objectives, Affymetrix Axiom was selected as a platform for the array.

### Content targeted for coverage

Content was selected for inclusion on the array in a modular fashion and then prioritized to create the final specifications for manufacturing. The content was compiled from multiple sources, including pathway and functional databases, NIDA, NIH-funded investigators, prior genotyping and sequencing projects, Affymetrix, and scientific publications.

#### Genome-wide association markers

A catalog panel was provided by Affymetrix to provide baseline genome-wide coverage across AFR, ASN, and EUR ancestry groups for discovery as well as meta-analysis across studies. 246,038 of the catalog markers overlap with the Affymetrix Axiom Biobank array. We included an additional panel of 50,000 markers provided by Affymetrix to increase genome-wide coverage in African ancestry populations, thereby maximizing genotype imputation efficiency to a larger variant set [74, 75].

#### Addiction-related genes

One thousand fourteen genes were identified as related to addiction through expert nomination, and recent bioinformatics projects and knowledge-bases. Table 4 presents the source, annotation, and gene count. Sources include the NIDA Genetics Consortium [76, 77], Gene Ontology [78], QIAGEN’s Ingenuity Pathway Analysis [79], and Pharmacogenetics of Nicotine Addiction Treatment [31, 37].

**Table 4:** Smokescreen addiction genes: source, categories, and counts*

Source	Category	Genes
Gene Ontology	dopamine_receptor_binding	8
Gene Ontology	dopamine_binding	9
Gene Ontology	serotonin_uptake	4
Gene Ontology	serotonin_metabolic_process	8
Gene Ontology	serotonin_transport	12
Gene Ontology	response_to_nicotine	31
Gene Ontology	dopamine_secretion	15
Gene Ontology	dopamine_uptake	8
Gene Ontology	dopamine_receptor_signaling_pathway	30
Gene Ontology	dopamine_transport	23
Gene Ontology	serotonin_secretion	8
Gene Ontology	dopamine_metabolic_process	26
Gene Ontology	regulation_of_dopamine_secretion	15
IPA	cigarette_habituation_susceptibility_syndrome	6
IPA	nicotine	14
IPA	susceptibility_to_drug_addiction	1
IPA	addiction_behavior	24
IPA	tobacco	27
IPA	addiction	131
IPA	withdrawal	11
IPA	smoking	12
IPA	naltrexone	4
IPA	clonidine	7
IPA	nortripyline	3
IPA	varenicline	3
IPA	nicotine	19
IPA	bupropion	3
NIDA Genetics Consortium	The Nicotine System	20
NIDA Genetics Consortium	The Dopamine System	10
NIDA Genetics Consortium	Mouse QTL	423
NIDA Genetics Consortium	The Alcohol System	32
NIDA Genetics Consortium	The Cholinergic System	9
NIDA Genetics Consortium	The Adrenergic System	16
NIDA Genetics Consortium	Tyrosine	3
NIDA Genetics Consortium	Other	263
NIDA Genetics Consortium	The GABA System	31
NIDA Genetics Consortium	Neurotransmitter Transporters	13
NIDA Genetics Consortium	The Nicotine Metabolism System	11
NIDA Genetics Consortium	The Serotonergic System	20
NIDA Genetics Consortium	The Endocannabinoid System	2
NIDA Genetics Consortium	Dopamine Synthesis	2
NIDA Genetics Consortium	The Glutamatergic System	42
NIDA Genetics Consortium	The Opioid System	12
PNAT		134

*Genes in categories may overlap

#### Fine mapping of smoking-related loci

We aimed for the densest coverage of any genotyping array for the chr15q25.1 nicotinic acetylcholine receptor (nAChR) gene cluster (*CHRNA5*, *CHRNA3* and *CHRNB4),* and the chr19q13.2 nicotine metabolizing enzyme genes (*CYP2A6* and *CYP2B6)*. The *CYP2A6* gene plays a major role in the nicotine metabolism pathway [80, 81] while genes encoding for CYP isozymes, such as the *CYP2B6* gene, may play a smaller role in influencing nicotine metabolism [82, 83]. An individual’s nicotine metabolism affects the level of circulating and sequestered nicotine and thus, nicotine intake [40, 84]. Nicotine binds to nAChRs, triggering neurotransmitter release and leads over time to nicotine dependence. nAChR activity, and thus nicotine dependence, is regulated by the cholinergic genes on chromosomes 8p11.21, 15q25.1 and 20q13.33 [16, 17, 21, 85–88].

### Filtering and tagging of selected markers

Each content category was submitted to Affymetrix as a list of markers or genomic regions. Affymetrix used proprietary software and a Axiom-validated marker database to select the best-performing markers (one or more probesets per marker) that covered the targeted content, either through direct inclusion or through efficient pairwise tagging. Multiple probesets were selected for markers that are either non-validated or deemed high priority (e.g., markers with known associations with addiction, smoking behavior, or nicotine metabolism), in order to minimize genotyping failures of these markers.

Using genotype data from the 1000 Genomes Project (Phase 1, March 2012 release), in African (YRI), East Asian (CHB + CHS + JPT) and European (CEU + FIN + GBR + IBS + TSI) populations, tagging the 1014 addiction-related genes (±20 kb) was performed in three rounds: (1) all markers with an MAF ≥ 0.05 were tagged at *r*^2^ ≥ 0.9 using Axiom-validated markers only; (2) remaining untagged markers were tagged at *r*^2^ ≥ 0.8 using Axiom-validated markers only; (3) any remaining untagged markers were tagged at *r*^2^ ≥ 0.9 using non-validated markers.

Both the range encompassing the entirety of *CYP2A6* and *CYP2B6* genes (±20 kb) and the range defined by the largest linkage disequilibrium (LD) block encompassing the nAChR gene cluster were defined. All known variants were selected from multiple databases, including 1000 Genomes Project Phase 1, HapMap, and the NHLBI GO Exome Sequencing Project [89].

### Imputed coverage estimation

Imputation coverage was estimated for the Smokescreen array using an approach similar to that described by Nelson et al. [62]. 1000 Genomes Project Phase 1 data (March 2012 release) were extracted for the Smokescreen content as an imputation inference set. All 1000 Genomes Project Phase 1 SNPs were used as the reference set. For each population (EUR, ASN, YRI), groups of 10 samples were created. For each group, the samples were kept in the inference set and excluded from the reference set, and imputed separately from other groups. Samples with known or cryptic relatedness with other samples in the 1000 Genomes Project were excluded both from the inference set groups and reference sets. Beagle version v4.0 release 1230 was used for phasing and imputation of chromosomes 1 to 22 with default settings [90]. Imputation was broken up by chromosome and results were then combined for all SNPs and groups of samples. For each SNP, we computed the correlation between the imputed dosages and measured genotypes from the 1000 Genomes Project (obsRSQ). The obsRSQ were then summarized overall (genome-wide), for the addiction-genes and the fine-mapping regions for each population.

### Smokescreen genotyping pilot

DNA samples from 188 Hapmap and 1000 Genomes Project participants from CEPH/Utah, Yoruba in Ibadan, Nigeria, and Japanese in Tokyo, Japan populations were obtained from Coriell Institute for Medical Research (Camden, NJ); 442 study samples from NIDA-funded Investigators, and six positive controls provided by the genotyping lab, were obtained for the genotyping pilot (see Table 5).

**Table 5:** Smokescreen pilot genotyping sample characteristics - blood or cell line

Study	Description	Samples	Individuals	DNA Source	%Male
Hapmap and 1000 Genomes	CEPH/Utah; Yoruba in Ibadan, Nigeria; Japanese in Tokyo, Japan samples	188	188	cell line	50.00 %
PKTWIN, “588”, SMOFAM	Nicotine metabolism laboratory studies	343	326	blood	45.71 %
Total Exposure Study	Cross sectional study of tobacco exposures	33	32	blood	56.25 %
MA fMRI, Modafinil, ASCC2, ASCC Neural Systems	Genetics and brain structure in smokers	35	34	blood	58.82 %
COGEND	Report of genetic results in smokers	31	30	blood	33.33 %
Positive lab controls		6	1	cell line	

#### Ethics approval and consent to participate

All individuals provided written informed consent. The Collaborative Genetic Study of Nicotine Dependence (COGEND) study was approved by the Washington University Institutional Review Board. The Early Methamphetamine Abstinence: fMRI and Cognition (MA fMRI); Neural Systems, Inhibitory Control, and Methamphetamine Dependence (Modafinil); Neural Systems and Risk for Adolescent Smoking (ASCC2); and Neural Correlates of Cognition, Craving and Reward Delay in Adolescent Smokers (ASCC Neural Systems) studies were approved by the University of California, Los Angeles Institutional Review Board. The Total Exposure Study (TES) was approved by the SRI International Human Subjects Committee. The Pharmacokinetics in Twins (PKTWIN), Pharmacogenetic Study of Nicotine Metabolism (588), and Smoking in Families (SMOFAM) studies were approved by the committee on Human Research at the University of California San Francisco and the Human Subjects Committee at SRI International.

A total of 636 purified DNA samples (including 27 replicates) derived from blood or cell line were processed on the Smokescreen Genotyping Array at RUCDR Infinite Biologics (Piscataway, NJ) according to manufacturer instructions. Raw data consisted of one CEL file per sample (except for one sample that failed to scan). Following best practices guidelines set forth by the manufacturer [55], the Affymetrix Power Tools (APT) v1.17.0 software was used to process raw data. Dish QC (DQC) values were generated and used to remove samples with DQC < 0.82. As part of stage 1 genotyping, 20,000 probesets previously validated by the manufacturer were used to cluster genotypes and remove samples with stage 1 call rate <97 %. Plate pass rate and average stage 1 call rate per plate were calculated and reviewed to determine if any plates should be excluded from further analysis. Remaining samples were genotyped for all probesets (stage 2 genotyping), using APT. The Affymetrix SNPolisher v1.5.2 software was used to classify probeset quality and determine the best probeset for each marker. Markers whose best probeset classified as “Other”, “CallRateBelowThreshold”, “Off-target variant (OTV)”, “Hemizygous”, or “MonoHighResolution” (or had previously failed Affymetrix’ internal validation process) were removed. A smaller number of DNA samples derived from saliva were also genotyped and clustered separately but are excluded here. Prior to statistical analysis, samples with sex discrepancies (reported versus expected), unexpected relatedness (half-sib or greater), and replicate samples with the lowest call rate were excluded. All statistical analyses were performed in [R] [91].

## Additional file

Additional file 1:
**Smokescreen addiction regions and estimated imputed coverage.** This file contains the addiction-related gene regions (chr 1–22) represented on the Smokescreen array and the estimated imputation-based coverage for each region in European (EUR), Asian (ASN), and African (YRI) populations. (XLSX 338 kb)

## Abbreviations

ADME: Absorption, distribution, metabolism, and excretion
AFR: African
AIM: Ancestry informative marker
APT: Affymetrix Power Tools
ASN: Asian
CEU: Utah Residents (CEPH) with Northern and Western European Ancestry
CHB: Han Chinese in Beijing, China
CHS: Southern Han Chinese
CPD: Cigarettes per day
DQC: Dish QC
EUR: European
FIN: Finnish in Finland
FTCD: Fagerström Test for Cigarette Dependence
GBR: British in England and Scotland
GWAS: Genome-wide association study
IBS: Iberian Population in Spain
IPA: Ingenuity Pathway Analysis
JPT: Japanese in Tokyo, Japan
LD: Linkage disequilibrium
MAF: Minor allele frequency
nAChR: Nicotinic acetylcholine receptor
NIDA: National Institute on Drug Abuse
NMR: Nicotine Metabolite Ratio
NRT: Nicotine Replacement Therapy
OTV: Off-target variant
PNAT: Pharmacogenetics of Nicotine Addiction Treatment
SBIR: Small Business Innovative Research
SNP: Single nucleotide polymorphism
TSI: Toscani in Italia
YRI: Yoruba in Ibadan, Nigeria
